# Highly integrated, alignment healing, and full‐recyclable multifunctional flexible devices based on dynamic covalent bonds

**DOI:** 10.1002/smo2.70071

**Published:** 2026-06-24

**Authors:** Xianfei Cao, Yong Qi, Wenbin Niu, Bingtao Tang, Shufen Zhang

**Affiliations:** ^1^ State Key Laboratory of Fine Chemicals Frontier Science Center for Smart Materials Dalian University of Technology Dalian China; ^2^ College of Materials and Chemical Engineering Key Laboratory of Inorganic Nonmetallic Crystalline and Energy Conversion Materials China Three Gorges University Yichang China

**Keywords:** alignment healing, dynamic covalent bonds, layer‐by‐layer integration, multifunctional flexible devices, recyclable

## Abstract

Integrated multifunctional flexible devices are essential technologies in intelligent sensing; however, their development is hindered by challenges such as unstable device interfaces, difficulties in precise damage repair, and limitations in material recovery. This study proposes a strategy of constructing functional layers alternately using crosslinked polymers with incompatible backbones but sharing the same dynamic bonds. This design utilizes identical dynamic covalent bonds to achieve strong interlayer bonding, approaching the strength of bulk materials. The incompatible backbones enable self‐alignment and precise healing of functional layers, while the dynamic bonds confer recyclability. Based on this approach, we successfully fabricated highly interface‐stable optoelectronic sensors incorporating nanospheres, conductive media, and magnetic particles. Following damage, these sensors self‐assembled under magnetic guidance and regained functionality after thermal treatment. Furthermore, the dynamically crosslinked polymer in the device dissolves upon heating, allowing for the complete recovery of both polymer and functional particles based on the distinct physical properties of the nanoparticles. This work simultaneously addresses the challenges of integration, repair, and recycling in flexible multifunctional devices through the design of dynamic bond materials, offering insights for their advancement.

## Graphical abstract

This study addresses stability, repair, and recycling in flexible devices using polymers with identical dynamic bonds but incompatible backbones. This enables robust integration, precise self‐healing, and full recyclability. We demonstrate optoelectronic sensors that, after damage, magnetically self‐assemble and heal upon heating. Subsequently, the dynamic network dissolves, recovering all components. This material strategy advances sustainable multifunctional electronics.

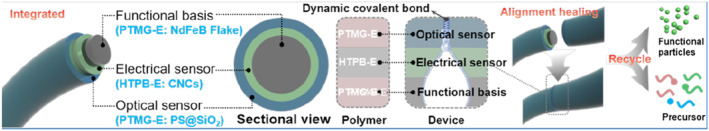

## 1 INTRODUCTION

The rapid advancement of technology has unveiled significant potential for highly integrated, flexible multifunctional devices in areas such as health monitoring,[^1^, ^2^, ^3^, ^4^, ^5^] biomedicine,[^6^, ^7^] and self‐powered smart wearables.[^8^, ^9^, ^10^, ^11^, ^12^] Traditional integration of multifunctional components often relies on methods like interfacial adhesion,[^13^, ^14^] Janus structures,[^15^, ^16^, ^17^] and physical lamination.[^18^, ^19^] Enhancing the interfacial adhesion between functional layers is crucial for improving the overall stability and reliability of these multifunctional devices.^[20]^ Strategies employing hydrogen bonds[^21^, ^22^, ^23^] and coordination interactions[^24^, ^25^, ^26^] have proven effective in boosting interlayer stability. Bao et al.[^27^, ^28^] utilized dynamic urea bonds to integrate multifunctional components, constructing flexible devices capable of stable operation even when subjected to high temperatures, underwater conditions, and stretching. This further demonstrates that dynamic covalent bonds hold significant application potential in the field of integrated high‐performance devices.

The ability for multilayered, flexible multifunctional devices to recover from various damages—such as punctures, scratches, and cuts—during use would significantly enhance their durability and service life.^[29]^ However, as the complexity of integrated devices increases, achieving synchronous alignment healing across multiple, distinct functional layers has become a critical performance requirement for highly integrated flexible systems. The manual realignment of different functional components after damage is highly impractical for ultra‐thin devices.^[30]^ Even minor misalignment between layers can prevent the restoration of functionality. This fundamental issue stems from the use of a single type of alignment healing polymer for the entire device. While using an identical polymer ensures strong interlayer adhesion, it fails to provide selective driving forces for different functional components during the healing process, thereby preventing precise realignment. Bao et al.^[27]^ demonstrated a multilayer alignment healing device composed of two self‐healing polymers that share identical dynamic bonds but feature immiscible polymer backbones. When misalignment occurs after damage, these multilayer structures reconfigure through a process of composition gradient‐driven chain diffusion. The subtle interdiffusion at the polymer interfaces also effectively prevents the diffusion of embedded particles, preserving the electronic functionality of each layer and avoiding mixing induced by damage. On another front, dynamic covalent bond‐based polymers exhibit excellent recyclability and reprocessability.[^31^, ^32^, ^33^, ^34^] Given the high cost of functional particles used in these devices, the ability to recover these particles and recycle the flexible matrix from discarded devices would substantially reduce material waste. Dynamic covalent bond materials demonstrate significant application potential across the domains of device integration, damage repair, and recyclability. Through rational polymer design and device integration, it is possible to realize multifunctional flexible devices that feature robust integration, independent healing of functional layers, and full recyclability. This approach promises to establish a new paradigm for the fabrication of next‐generation intelligent devices and the selection of their constituent materials.

**FIGURE 1:**
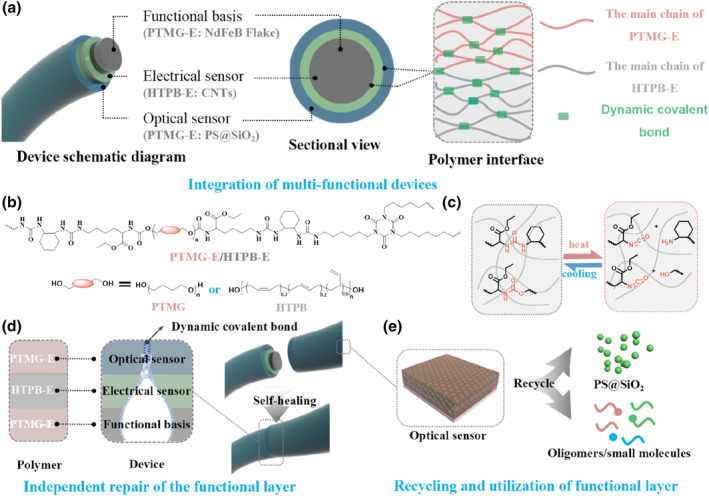
Schematic diagram of the highly integrated, alignment‐healing, and fully recyclable multifunctional flexible devices based on dynamic covalent bonds. (a) Schematic of the integrated optoelectronic device based on dynamic bonds. (b) Molecular structures of the dynamic urethane‐urea elastomers. (c) Exchange mechanism of the dynamic urethane‐urea bonds. (d) Schematic of the healing process of the dynamic‐bond‐based optoelectronic device. (e) Schematic of the recycling process.

Herein, we design and synthesize two new dynamic elastomers, PTMG‐based elastomer (PTMG‐E) and HTPB‐based elastomer (HTPB‐E), based on poly (trimethylene glycol) (PTMG) and hydroxy‐terminated polybutadiene (HTPB), respectively. Although their backbones are thermodynamically incompatible, both elastomers incorporate the same dynamic urethane‐urea bonds. This molecular design enables the fabrication of highly integrated multifunctional flexible devices that exhibit alignment and repair after damage, together with full recyclability. The shared dynamic covalent bonds ensure strong interfacial fusion between PTMG‐E and HTPB‐E layers, yielding integrated devices with high structural integrity and robustness. Meanwhile, the inherent backbone incompatibility preserves functional independence and confers a unique self‐alignment capability during healing (Figure 1). When a fractured device is magnetically reassembled and thermally treated, the functional layers realign and repair via dynamic bond exchange, restoring photodetector performance to nearly its original level. Furthermore, the dynamic cross‐links allow complete recycling of the devices. Through a designed recycling process, both the functional nanoparticles and the polymer matrix can be fully recovered. The reclaimed nanoparticles retain their original morphology and properties, while the recycled polymer films maintain excellent mechanical performance. Dynamic bond chemistry offers a material design approach for flexible device integration, damage repair, and recycling, establishing a new paradigm for designing next‐generation flexible devices.

## 2 RESULTS AND DISCUSSION

### 2.1 Synthesis of polymers based on dynamic covalent bonds

**FIGURE 2:**
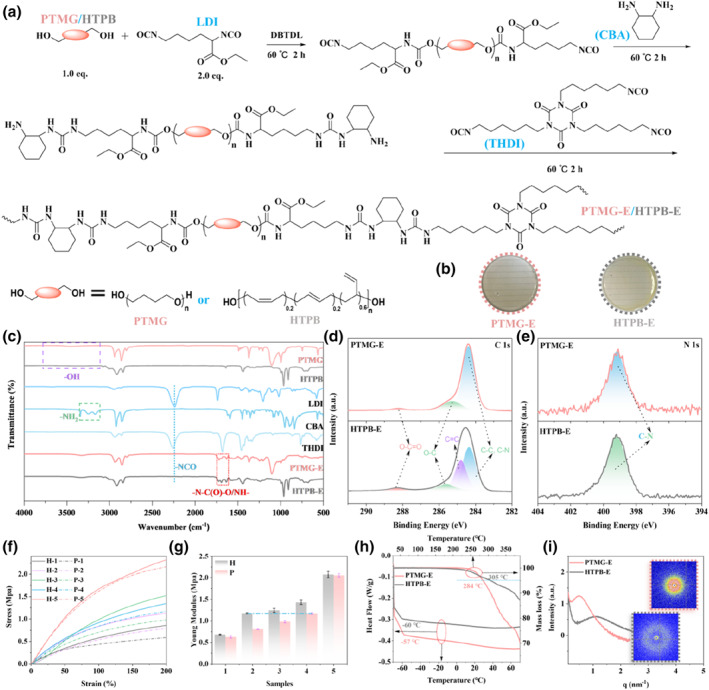
Preparation and characterization of PTMG‐E/HTPB‐E. (a) Chemical structures of elastomers. (b) Optical photograph of PTMG‐E/HTPB‐E (Sample size: 10 cm diameter). (c) FT‐IR spectra of precursors and PTMG‐E/HTPB‐E. XPS C 1s (d) and N 1s (e) plots of PTMG‐E and HTPB‐E. (f) Stress‐strain curves of HTPB‐based and PTMG‐based elastomers at 200% strain under different mixing ratios. (g) Young’s modulus of HTPB‐based and PTMG‐based elastomers. (h) TGA and DSC curves of PTMG‐E and HTPB‐E. (i) SAXS curves showing the amorphous structure of PTMG‐E and HTPB‐E.

For the molecular design of immiscible dynamic polymers, backbone segments combining transparency, flexibility, and integration capability were essential. Therefore, poly (tetramethylene glycol) (PTMG) and hydroxyl‐terminated polybutadiene (HTPB) were chosen. A crosslinked poly (urethane‐urea) material (Figure 2b) was synthesized with diols (PTMG/HTPB) as soft segments, 1,2‐diamino‐cyclohexane (CBA) as a chain extender, and trimerized hexamethylene diisocyanate (THDI) as a crosslinking agent (Figure 2a). The Fourier Transform Infrared spectrum revealed strong absorption bands for HTPB and PTMG in the 3300 to 3500 cm^−1^ region, attributed to the stretching vibration of ‐OH groups. CBA exhibited characteristic primary amine peaks at 3100 to 3300 cm^−1^; LDI and THDI showed characteristic ‐NCO peaks at 2250 to 2260 cm^−1^. These characteristic peaks disappeared in the post‐reaction PTMG‐E and HTPB‐E. A new peak at ∼1700 cm^−1^ corresponding to ‐NH‐CO‐O/NH‐CO‐NH‐ indicates the formation of the crosslinked polymer network^[35]^ (Figure 2c and Supporting Information S1; Figure S1, Supporting Information). Additionally, X‐ray photoelectron spectroscopy spectra revealed peaks for C and N elements at binding energies of 288.4 and 399.2 eV, corresponding to ‐O‐C=O and C‐N groups (Figure 2d,e), respectively, further indicating the formation of a poly(urethane‐urea) crosslinked network.

For practical application considerations, we evaluated the tensile strength and Young's modulus of different PTMG‐ and HTPB‐based elastomers at 200% strain (Figure 2f,g and Supporting Information S1; Figure S2a,b). The H‐2 and P‐4 formulations (H‐2: HTPB: LDI: CBA: THDI = 1: 2: 1.2: 0.07, P‐4: PTMG: LDI: CBA: THDI = 1: 2: 1.6: 0.21, Supporting Information S1; Tables S1, Supporting Information) exhibited closely matched tensile strength and Young's modulus within the 200% strain range. Therefore, they were selected as the flexible substrates for subsequent device integration, ensuring compatible mechanical properties across layers. Consequently, subsequent studies focus on these two elastomers, referred to as HTPB‐E and PTMG‐E for brevity. Simultaneously, PTMG‐E and HTPB‐E demonstrated low glass transition temperatures (<−55°C) and high thermal stability (>280°C) (Figure 2h), indicating their suitability for applications over a wide temperature range.

Small‐angle X‐ray scattering confirmed the absence of larger microphase‐separated structures, with a characteristic domain spacing between 12.92 and 5.67 nm[^36^, ^37^] (Figure 2i). This nanoscale microphase separation indicates that while the PTMG and HTPB segments are thermodynamically immiscible, their phase separation is constrained. This confined nanostructure provides an ideal environment for molecular chain movement and dynamic exchange at the interfaces. Therefore, the composite formation and healing process between the two immiscible polymers is predominantly governed by molecular chain dynamics at the interfaces.^[27]^

### 2.2 Interfacial and repair behavior of two immiscible dynamic polymers

**FIGURE 3:**
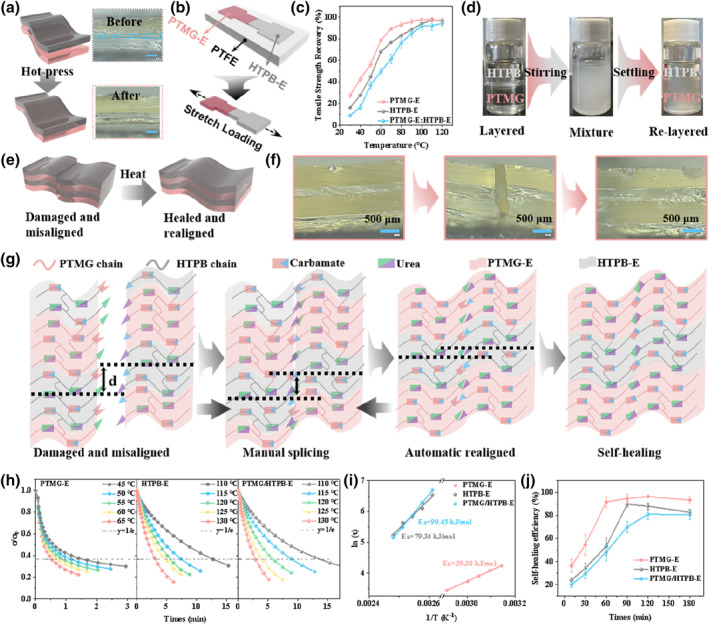
Interface and healing behavior of PTMG‐based elastomer (PTMG‐E) and HTPB‐based elastomer (HTPB‐E) polymers. (a) Microscopic images of PTMG‐E and HTPB‐E before and after hot pressing (Scale bar: 500 μm). (b) Schematic of interface bonding and tensile testing for semi‐dumbbell‐shaped PTMG‐E and HTPB‐E specimens, and (c) the recovery in tensile strength. (d) Digital photograph of hydroxy‐terminated polybutadiene (HTPB) and PTMG re‐lamination after mixing (Sample bottle: 10 mL). Schematic of fracture and self‐aligning repair in multilayer PTMG‐E/HTPB‐E composites (e), Optical microscopic image (Scale bar: 500 μm) (f), and schematic of the mechanism (g). (h) Stress relaxation curves and (i) Arrhenius activation energy analysis for PTMG‐E, HTPB‐E, and PTMG/HTPB‐E. (j) Self‐healing efficiency of PTMG‐E, HTPB‐E, and PTMG/HTPB‐E after different curing times at 70°C (*η* = elongation at break after healing/original elongation at break).

PTMG‐E and HTPB‐E share identical dynamic bonds, which enabled the formation of a robust interface between them. A two‐layer composite was laminated via mild hot‐pressing (∼20 kPa, Figure 3a). The polymer interface was examined using a super‐depth‐of‐field optical microscope before and after annealing at 70°C. After hot‐pressing, an interface with no discernible gap was formed. To further evaluate the interfacial bond strength, semi‐dumbbell specimens of each polymer were prepared and joined. Following thermal treatment at different temperatures for 30 min, tensile tests were conducted (Figure 3b). With increasing treatment temperature, the tensile strength of the composite film approached that of the individual bulk polymer films (Figure 3c). This demonstrates that a stable, dynamically cross‐linked interface can be formed between PTMG‐E and HTPB‐E after a simple thermal treatment (e.g., 70°C for 30 min).

The main prerequisite for self‐aligning healing in multilayer polymer films is the incompatibility of polymer segments. We first tested the mixing and re‐stratification capability of PTMG and HTPB to verify the incompatibility of the main chains in PTMG‐E and HTPB‐E by sequentially adding appropriate amounts of PTMG and HTPB into sample vials, stable phase interfaces formed between the two prepolymers. After mixing, stirring, and settling, HTPB and PTMG rapidly separated into distinct layers, demonstrating their excellent incompatibility (Figure 3d). Through transmittance measurements, dye diffusion experiments, and Atomic Force Microscopy modulus mapping of HTPB‐E and PTMG‐E, and their composite elastomer (HTPB/PTMG‐E), it can be seen that PTMG‐E and HTPB‐E exhibit pronounced immiscibility (Supporting Information S1; Figure S2c–f). Leveraging the incompatibility of PTMG‐E and HTPB‐E, a complete film with four alternating layers of PTMG‐E and HTPB‐E was fabricated. Upon cutting the resulting film, as shown in Figure 3e,f, dislocation phenomena were observed between the alternating layers. During the alignment healing process, each layer rearranged, forming distinct and alternating interfaces between PTMG‐E and HTPB‐E. The initial misalignment on the polymer surface (marked as d) progressively achieves precise alignment through selective diffusion and mutual diffusion after material fusion, ultimately reaching a fully ordered state^[27]^ (Figure 3g).

It was hypothesized that dynamic bond exchange occurs more readily within the same polymer, making healing between homopolymers less challenging and thereby facilitating self‐organized healing between different polymers. To validate this hypothesis, we tested the stress relaxation behavior of PTMG‐E, HTPB‐E, and the crosslinked elastomer (PTMG/HTPB‐E) prepared by blending PTMG and HTPB at different temperatures (Having an equal amount of dynamic covalent bonds), and analyzed their relaxation activation energies accordingly (Figure 3h,i). Results indicate that PTMG/HTPB‐E exhibits higher relaxation activation energy (Ea = 99.43 kJ/mol) than either PTMG‐E or HTPB‐E alone. This may stem from microphase separation occurring during the blending and crosslinking of PTMG and HTPB, which generates numerous polymer interfaces during dynamic bond exchange, thereby increasing the difficulty of bond transfer.^[38]^ To further validate this hypothesis, we evaluated the self‐healing performance of PTMG‐E, HTPB‐E, and PTMG/HTPB‐E. After cutting the samples, thermal healing experiments were conducted at 70°C for varying durations, and healing efficiency was assessed via stress‐strain curves (Figure 3j). Results indicate that PTMG/HTPB‐E exhibits lower self‐healing capability than PTMG‐E and HTPB‐E. This suggests that healing is more challenging in blended polymer systems, where healing tends to occur within a single phase rather than across phases. This phenomenon primarily stems from easier segment diffusion within homophases, which facilitates alignment healing and healing in multilayer polymers.

### 2.3 Integration and functional healing of multifunctional flexible devices

**FIGURE 4:**
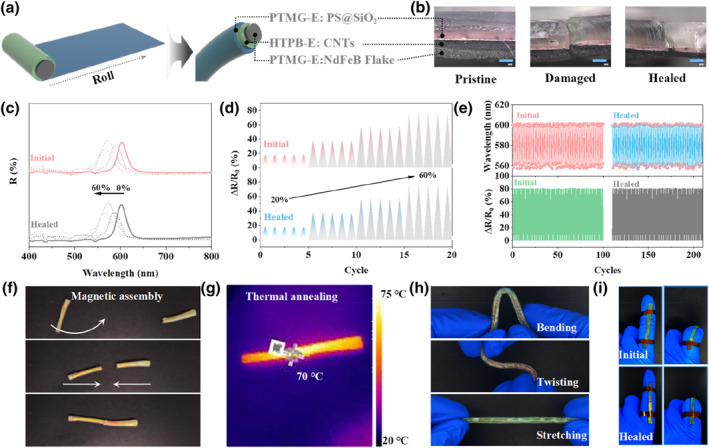
Study on the integration and self‐healing performance of multifunctional flexible devices. (a) Schematic of multifunctional flexible device fabrication via roll‐to‐roll process. (b) Optical image of self‐aligned repair in multifunctional devices (Scale bar: 500 μm). (c) Optical performance and (d) electrical performance before and after device repair (70°C for 30 min), along with (e) durability testing. (f) Photograph of magnetically controlled assembly and (g) 70°C treatment of multifunctional flexible devices. (h) Images of the welded device bending, twisting, and stretching to show mechanical robustness (Device dimensions: diameter: 4 mm; length: 60 mm). (i) Photograph of the optical sensor device for finger bending before and after curing.

To demonstrate that alternating layers of immiscible dynamic polymers promote alignment during the repair of thin (approximately 10–100 μm) multilayer electronic devices, we investigated the functional repair of multifunctional flexible devices. We employed homogeneous composite layers of PTMG‐E embedded with magnetic particles NdFeB (80% by weight), HTPB‐E homogeneous composite layers with conductive carbon nanotubes (CNTs, 10% by weight), and PS@SiO_2_ microsphere‐based photonic crystal array composite PTMG‐E mechanochromic composite film. This layered roll‐to‐roll process yielded a flexible fiber sensor integrating a functional magnetic core, electrical response, and mechanochromic properties (Figure 4a). Figure 4b displays microscopic images of the fiber composite device cross‐section in pristine, damaged, and repaired states. Despite misalignment after damage, the distinct functional layers of the multilayer flexible device realigned during repair and restored sensing capability, exhibiting quantitatively similar optical and electrical sensing performance under identical strain conditions (Figure 4c,d). We also monitored the cyclic performance of the device's optoelectronic properties before and after repair (Figure 4e). The cyclic optoelectronic response of the repaired multifunctional flexible device closely matched that of the pristine device, attributed to the realignment of functional layers alongside rapid repair.

The fibrous functional device was magnetized along its longitudinal axis using a pulsed magnetizer (1.5 T), imparting magnetic properties to the NdFeB‐PTMG‐E core material. When cut into small pieces, the movement of the fibers can be controlled by an external magnetic field, enabling rigid body rotation for self‐reassembly without any manual alignment. When brought into proximity, these fibers exhibit magnetic attraction, generating contact pressure that facilitates selective layer welding (Figure 4f and Supporting Information S2; Video S1). Following thermal welding at 70°C (Figure 4g), the magnetically assembled fiber devices can withstand deformations such as bending, twisting, and stretching (Figure 4h). Unlike single‐component magnetic self‐healing technologies, which enable macroscopic assembly of components but lack precision in microscopic alignment, this study demonstrates the simultaneous utilization of two alignment mechanisms: magnetically guided macroscopic alignment and interfacial tension‐mediated microscopic alignment. Application of multifunctional flexible sensors for detecting human finger bending motion. As the finger bends, the sensor's color changes from orange‐red to yellow‐green (Figure 4i), with the corresponding spectral reflection wavelength shifting from 605 to 560 nm (Supporting Information S1; Figure S3, Supporting Information). Meanwhile, the electrical signal exhibited regular variations in response to rhythmic finger bending (Supporting Information S1; Figure S4, Supporting Information). After magnetic‐controlled assembly and heat treatment, the severed device retained excellent sensing capability, as shown in Figure 4i and Supporting Information S1; Figure S5.

### 2.4 Recycling of multifunctional flexible devices based on dynamic bonds

**FIGURE 5:**
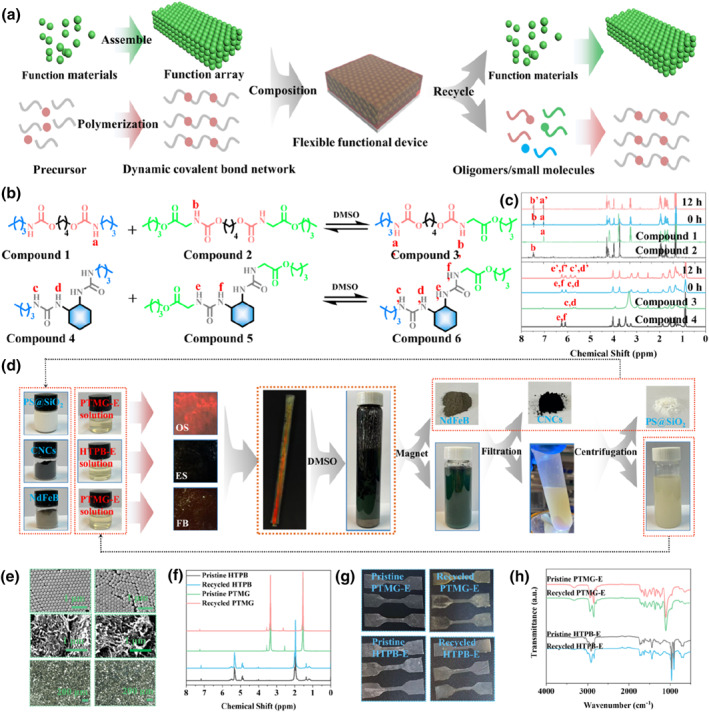
Recycling of flexible functional devices and mechanisms. (a) Schematic of the preparation and recycling process for dynamic‐bond‐based flexible multifunctional devices. (b) Schematic of the exchange reaction between Compounds 1 and 2 and between Compounds 4 and 5. (c) ^1^H NMR spectra indicating the formation of new exchange products, Compounds 3 and 6. These spectra were recorded after 12 h of reaction at 150°C. (d) Schematic of the preparation process for flexible functional devices based on dynamic (urethane‐urea) bonds and the recycling flowchart for functional particles and polymers. (e) Microstructural morphology of PS@SiO_2_ microspheres, CNTs, and NdFeB particles before and after recovery (left: pristine, right: recycled). (f) ^1^H NMR spectra of PTMG and HTPB monomers before and after recovery. (g) Digital photographs of PTMG‐E and HTPB‐E before and after recovery. (h) IR spectra of PTMG‐E and HTPB‐E before and after recovery. HTPB, hydroxy‐terminated polybutadiene; HTPB‐E, HTPB‐based elastomer; PTMG‐E, PTMG‐based elastomer.

Complete recyclability of flexible functional devices is crucial for advancing sustainable functional device development. In this study, the crosslinked network based on dynamic (urethane‐urea) bonds exhibits outstanding dissolution and recyclability. PTMG‐E can be completely dissolved by heating in dimethyl sulfoxide at 150°C for 10 min (Supporting Information S1; Figure S6, Supporting Information). Pouring the dissolved polymer solution into a mold and heating to remove the solvent yields a recycled polymer film with mechanical properties comparable to the pristine film (Supporting Information S1; Figure S7). Based on this, the multifunctional flexible devices constructed from the dynamic (urethane‐urea) bond‐based elastomer in this study demonstrate the potential for complete recovery of both functional particles and the polymer (Figure 5a). To validate the polymer's recyclability mechanism, small‐molecule model compounds were used to demonstrate the dynamic characteristics of the 1,2‐diaminocyclohexane‐urea bond and the urethane bond (Figure 5b). Considering the usage of LDI and THDI in polymers, compounds synthesized via reactions between butyl isocyanate (BI), isobutyl isocyanate acetate (IABE), 1,2‐diaminocyclohexane (CBA), and butanediol (BDO) were employed as model products. For instance, the reaction of BI with BDO yielded butane‐1,4‐diyl bis(butylcarbamate) (Compound 1), while the reaction of IABE with BDO produced dibutyl 4,11‐dioxo‐5,10‐dioxa‐3,12‐diazatetradecanedioate (Compound 2). These compounds underwent hydrogen NMR analysis at 150°C in DMSO‐d_6_ over varying time intervals (Figure 5c). New carbamate hydrogen peaks appeared in the hydrogen NMR spectra, confirming the occurrence of dynamic exchange reactions involving the carbamate bond.^[32]^ Model compounds 4 and 5 for the urea bond were subjected to hydrogen NMR analysis in DMSO‐d_6_ at 150°C over different time intervals (Figure 5c). New urea group hydrogen peaks appeared in the hydrogen NMR spectrum, confirming the occurrence of a dynamic exchange reaction for the urea bond.^[32]^

In this study, flexible multifunctional devices were fabricated by combining three functional particles—PS@SiO_2_, CNTs, and NdFeB—with two polymers, PTMG‐E and HTPB‐E, to form three functional device composites. The physical properties of the three functional particles and the soluble reZXC Xcovery characteristics of the polymers enable the recycling and reuse of both functional particles and polymers within the devices (Figure 5d). Immersion of the flexible multifunctional device in DMSO followed by heating at 150°C for 30 min induces gradual dissociation. The sample vial exhibits a bottom layer of NdFeB magnetic particles, an upper layer of black CNC particles, and an intermediate phase of PS@SiO_2_ polymer dispersion. Leveraging the magnetic response of NdFeB, the particles are magnetically fixed to the vial bottom. The upper layer containing CNTs and the PS@SiO_2_ polymer DMSO dispersion is separated. After washing and drying the isolated NdFeB particles, pure magnetic particles are obtained. The recovered magnetic particles retain excellent magnetic properties (Supporting Information S1; Figure S8). Filtration of the CNTs and PS@SiO_2_ polymer DMSO dispersion yields a CNC filter cake and a PS@SiO_2_ polymer DMSO filtrate. Washing and drying the CNC filter cake produces CNTs with similar properties to the original material (Supporting Information S1; Figure S9). Centrifugation separates the polymer dispersion from PS@SiO_2_ microspheres in the polymer DMSO dispersion of PS@SiO_2_. The PS@SiO_2_ microspheres obtained by centrifugation are washed with ethanol and dried to yield PS@SiO_2_ microspheres with good dispersibility and assembly capability (Supporting Information S1; Figure S10).

The recycling of polymers and the recovery of expensive functional particles will significantly advance the development of circular chemistry. The functional particles recovered in the aforementioned processes exhibit physical properties similar to those of the initial particles at the macroscopic level. SEM further characterized the microstructure of PS@SiO_2_ and CNT functional particles. As shown in Figure 5e, the morphology of PS@SiO_2_ and CNTs remained largely unchanged. When the recovered PS@SiO_2_ was redispersed in ethanol, it retained excellent assembly capability. The assembled photonic crystal templates exhibited vivid structural colors (Supporting Information S1; Figure S11). The tubular structure of the recovered CNTs remained intact. After dispersing them in a polymer system to fabricate electro‐responsive devices, their conductivity and responsiveness were comparable to those of devices using initial CNTs (Supporting Information S1; Figure S12, Supporting Information). Microscopic examination of NdFeB magnetic particles before and after recycling revealed no significant size change and retained good magnetizability. The recycled PTMG/HTPB‐E films maintained excellent mechanical properties, with elongation at break of 3600%, and tensile strength of 3.72 MPa (Supporting Information S1; Figure S13, Supporting Information). Alkali treatment of the recovered polymer solution disrupted the dynamic bonds within the polymer, releasing the parent PTMG and HTPB monomers. NMR characterization of the purified PTMG and HTPB confirmed that the chemical structure of the monomers remained unchanged before and after recovery, with a recovery rate exceeding 90%. The IR characterization of the elastomers prepared (Figure 5g) from recycled PTMG and HTPB further confirmed that the chemical structure of the polymer films remained almost unchanged (Figure 5h).

## 3 CONCLUSION

In summary, this study leveraged the incompatibility between PTMG and HTPB main chains to construct two polymers with superior interfacial bonding strength and alignment healing capabilities by introducing identical dynamic carbamate‐urea covalent bonds. When applied to fabricate flexible dual‐response magnetic core‐photovoltaic devices, the high interfacial integrity ensured multifunctional stability. Following a device fracture, the cross‐section reconnects under magnetic field guidance. Leveraging the main chain's incompatibility and dynamic bond exchange properties, a self‐aligning healing mechanism is synergistically achieved, enabling precise realignment of functional layers and restoration of optoelectronic sensing capabilities with performance comparable to the original device. Additionally, the dynamic covalent bonds confer exceptional recyclability to the crosslinked network. This study achieves the first fully closed‐loop recycling of multifunctional flexible devices, with recovered functional particles, monomers, and polymers exhibiting physical and chemical properties highly consistent with the original materials. This research establishes a material design paradigm for the integration, damage repair, and recycling of multifunctional flexible devices, while also pioneering new pathways for dynamic covalent chemistry applications in flexible electronics.

## CONFLICT OF INTEREST STATEMENT

The authors declare no conflicts of interest.

## ETHICS STATEMENT

There is no animal testing involved in this job, and thus no requirement for an ethics statement.

## Supporting information

Supporting Information S1

Video S1

## Data Availability

The data that supports the findings of this study are available in the supplementary material of this article.
