# Supplementary material for: Highly integrated, alignment healing, and full‐recyclable multifunctional flexible devices based on dynamic covalent bonds
Source: Smart Mol. 2026 Jun 24:e70071. Online ahead of print. doi: 10.1002/smo2.70071 (PMC13398629; doi:10.1002/smo2.70071)
Supplement: Supplementary file 1 — Supporting Information S1 [file SMO2-9999-0-s002.docx]

Supporting Information

**Highly Integrated, Alignment Healing, and Recyclable Multifunctional Flexible Devices Based on Dynamic Covalent Bonds**

Xianfei Cao^[a]^, Yong Qi^[b]^, Wenbin Niu^[a]^, Bingtao Tang^[a]^, and Shufen Zhang^[a]^*

[a] State Key Laboratory of Fine Chemicals, Frontier Science Center for Smart Materials, Dalian University of Technology, Dalian 116024, China

[b] College of Materials and Chemical Engineering, Key Laboratory of Inorganic Nonmetallic Crystalline and Energy Conversion Materials, China Three Gorges University, Yichang 443002, China.
^*^ Corresponding author Email: zhangshf@dlut.edu.cn

***Materials***

Poly(tetrahydrofuran) (PTMG, Mn = 2000), lysine diisocyanate (LDI), cyclohexanediamine (CBA), dibutyltin dilaurate (DBTDL), vinyltriethoxysilane, carbon nanotubes (CNTs), and dimethyl sulfoxide (DMSO) were purchased from Aladdin Reagent. Hydroxyl-terminated polybutadiene (HTPB, Mn = 3000) and the trimer of hexamethylene diisocyanate (THDI) were purchased from Energy Chemical. Styrene, sodium dodecyl sulfate (SDS), potassium persulfate (KPS), aqueous ammonia, and sodium hydroxide were purchased from Tianjin Damao Chemical Plant. All salts and solvents were used as received without further purification.

***Preparation of PTMG-based and HTPB-based Elastomers***

PTMG and HTPB were first degassed under vacuum at 80 °C for 12 hours to remove moisture. Then, 1 mmol of PTMG or HTPB was weighed into a 25 mL flask, mixed with 10 mL of DMSO, and stirred uniformly at 60°C. Subsequently, 2 mmol of LDI and 1 wt% of DBTDL were added, and the reaction was allowed to proceed for two hours under a nitrogen atmosphere to form isocyanate (NCO)-terminated prepolymers. After that, varying amounts (1.0, 1.2, 1.4, 1.6, or 2.0 mmol) of CBA were added. The reaction system was further maintained at 80°C for 0.5 h, followed by the addition of THDI (0, 0.07, 0.14, 0.21, or 0.34 mmol) to form the cross-linked polymer. The resulting solution was poured into a mold and dried in an oven at 80°C for 24 hours to remove DMSO. The solvent was then completely removed under vacuum at 80°C for 48 hours, yielding the poly(urethane-urea) elastomer. These elastomers, prepared with different amounts of chain extender and cross-linker, were designated as P-1, P-2, P-3, P-4, P-5, H-1, H-2, H-3, H-4, and H-5, respectively.

***Preparation of PS@SiO_2_ Microspheres***

PS microspheres were synthesized via a classic emulsion polymerization method. In a 250 mL three-neck flask, a specified amount of sodium dodecyl sulfonate (serving as a soft template) was added, followed by 135 mL of deionized water. The mixture was kept at a constant temperature of 80 °C in an oil bath for 10 minutes. Then, 15 g of styrene monomer was added, and mechanical stirring was carried out at 250 rpm under continuous N₂ protection. After 15 minutes, 0.15 g of potassium persulfate was introduced to initiate polymerization. The reaction was stopped after 6 hours, and the resulting emulsion was transferred to a beaker and left open for one week to allow complete quenching of free radicals. The size of the polystyrene colloidal microspheres can be adjusted by varying the amount of the surfactant sodium dodecyl sulfonate. Next, 3 mL of the above-prepared PS emulsion was transferred into a 250 mL flask, followed by the addition of 57 mL of deionized water. The mixture was stirred at 500 rpm for 5 minutes, after which 4 mL of ammonia aqueous solution and 27 mL of deionized water were added, and stirring continued for another 15 minutes. Subsequently, 1.5 mL of vinyltriethoxysilane was introduced, and the reaction proceeded for 3–5 hours. The size of the microspheres can be controlled by adjusting the reaction duration.

***Fabrication of Optical Sensors***

The previously prepared PS@SiO_2_ microspheres were washed once with deionized water and three times with anhydrous ethanol. They were then dispersed in 10 mL of anhydrous ethanol and subjected to ultrasonic dispersion until uniform. The microsphere dispersion was spread onto a polytetrafluoroethylene template and heated at 40 °C for 15 minutes. After complete solvent evaporation, a brilliant opal photonic crystal template was formed. The opal photonic crystal template assembled on the polytetrafluoroethylene plate was heated to 40 °C, and the prepared precursor filling solution (PTMG-E) was slowly injected into the opal photonic crystal template. The assembly was vacuum degassed for 30 minutes in a vacuum drying oven and then cured in a 60 °C atmosphere for 2 h. Finally, it was dried overnight in a vacuum drying oven.

***Fabrication of Electrical Sensors***

Add 5wt% CNTs into the HTPB-E precursor solution and mix them uniformly under high-speed stirring. Transfer the mixed conductive elastomer precursor solution into a tetrafluoroethylene mold. After removing the solvent, transfer it to a vacuum drying oven for overnight vacuum drying.

***Preparation of Magnetic Cores***

NdFeB particles (80 wt%, 5 μm in diameter) were dispersed in a PTMG-E dispersion. The precursor solution containing these particles was then poured into a mold. After curing and shaping, the material was cut into rectangles using a cutting die. The rectangles were then manually rolled for 5 minutes on a Teflon sheet at 70 °C to promote layer bonding and homogenize the fiber shape. Finally, the fibers were annealed in an oven at 70 °C for 1 h and magnetized at room temperature using a custom pulse magnetizer with a pulsed magnetic field of 1.5 T.

***Integration of Multifunctional Flexible Fiber Sensors***

Using the aforementioned NdFeB/PTMG‑E composite magnetic material as the core, the CNTs/HTPB‑E electrical film and the PS@SiO₂/PTMG‑E optical film were sequentially wound on a Teflon plate at 70 °C to form a three‑layer fibrous device. The assembly was then placed in a constant‑temperature chamber at 70 °C for an additional 12 h to produce a stable multifunctional flexible fiber sensor.

The multifunctional flexible fiber sensor was cut into two segments, which were rotationally aligned and connected under a magnetic field. They were then heated to 70 °C using an infrared lamp and bonded for 10 min, with the process monitored by an infrared thermal imager, resulting in a reconstructed fiber capable of bearing load.

***Synthesis of Compound 1***

Butyl isocyanate (2 mmol) and butanediol (1 mmol) were dissolved in 1 mL of deuterated DMSO, followed by the addition of 1 μL of DBTDL as a catalyst. The reaction was carried out at 60 °C for 2 hours. The resulting product was compound 1. The reaction mixture was directly used for NMR testing.

***Synthesis of Compound 2***

2 mmol of n-butyl isocyanatoacetate and 1 mmol of butanediol were added to 1 mL of deuterated DMSO, followed by the addition of 1 microliter of DBTDL to catalyze the reaction. The mixture was reacted at 60 °C for 2 hours. The resulting product was compound 2. The reaction solution was used directly for NMR testing.

***Synthesis of Compound 4***

Butyl isocyanate (2 mmol) and CBA (1 mmol) were dissolved in 1 mL of deuterated DMSO. The reaction was carried out at RT for 2 hours. The resulting product was compound 4. The reaction mixture was directly used for NMR testing.

***Synthesis of Compound 5***

n-butyl isocyanatoacetate (2 mmol) and CBA (1 mmol) were dissolved in 1 mL of deuterated DMSO. The reaction was carried out at RT for 2 hours. The resulting product was compound 5. The reaction mixture was directly used for NMR testing.

***Recycling of Multifunctional Flexible Sensors***

Add the multifunctional flexible sensor to DMSO solvent at a mass ratio of 1:10, then heat and stir at 150 °C for 30 min. PTMG-E and HTPB-E dissolve, while the functional particles—NdFeB, PS@SiO₂, and CNTs—are released. Place the sample vial on a magnet to accelerate the settling of NdFeB particles, which become firmly fixed at the bottom of the vial due to magnetic attraction. Remove the upper mixed solution containing polymer/CNTs/PS@SiO₂. The separated NdFeB particles are washed with ethanol and dried. The polymer/CNTs/PS@SiO₂ mixed solution is filtered through filter paper (<10 μm); CNTs form a filter cake, which is washed with ethanol and dried. The filtrate is centrifuged at 8000 rpm for 10 min to obtain PS@SiO₂ solids and a dispersion of PTMG-E and HTPB-E. Pour off the PTMG‑E/HTPB‑E dispersion, wash the PS@SiO₂ with ethanol, and dry it. The PTMG‑E/HTPB‑E dispersion is poured into a mold to obtain a PTMG/HTPB‑E composite elastomer. To further recover PTMG and HTPB monomers, add 5 mL of 0.1 M sodium hydroxide solution to the PTMG‑E/HTPB‑E dispersion and continue stirring at 70 °C for 1 h. Then add 10 mL of tetrahydrofuran (THF) and 10 mL of water to induce phase separation. Separate the organic phase, wash it three times with water, and evaporate the THF using a rotary evaporator. After standing, the lower liquid layer is PTMG, and the upper layer is HTPB. Through the above steps, pure functional particles such as NdFeB, CNTs, and PS@SiO₂, as well as PTMG and HTPB monomers, can be recovered.

***Characterization:***

Infrared characterization was obtained using a Thermo Fisher iS50 Fourier Transform Infrared Spectrometer in reflection mode. The C1s and N1s spectra of the elastomers PTMG-E and HTPB-E were obtained through routine analysis using an X-ray photoelectron spectroscopy ESCALAB Xi+ from Thermo Fisher Scientific UK. The stress-strain curves of the elastomers were measured with a universal tensile testing machine PT-305B from Guangdong Beidou Precision Instrument Co., Ltd. Thermal characterization was performed using thermogravimetric analysis (TGA) and differential scanning calorimetry (DSC). The TGA curves were measured on a TA Instruments Q500 thermogravimetric analyzer, and the DSC curves were obtained using a TA Instruments Q2000 differential scanning calorimeter. Small-angle X-ray scattering (SAXS) measurements were performed using a Rigaku NANOPIX SAXS instrument with a sample-to-detector distance of 625 mm. Optical microscopy images of the samples were obtained using a Keyence VHX-7000N digital microscope (Japan) with ultra-depth-of-field observation capability. Stress relaxation experiments on the samples were conducted using the dual cantilever fixture of a TA Instruments Q800 dynamic mechanical analyzer. The optical and electrical signals of the sensor were measured using a PG2000-Pro fiber optic spectrometer (Shanghai Ideaoptics Corp., Ltd.) with a vertically incident light source, and an LCR meter (TH2833) operating at a voltage of 1 V and a sweep frequency of 1 kHz, respectively. NMR spectra were recorded on a BRUKER AVANCE NEO 400 MHz spectrometer using deuterated DMSO (DMSO‑d₆) as the solvent.

***Figures and Tables***

**Figure S1.** IR Spectra of Raw Materials and Elastomers


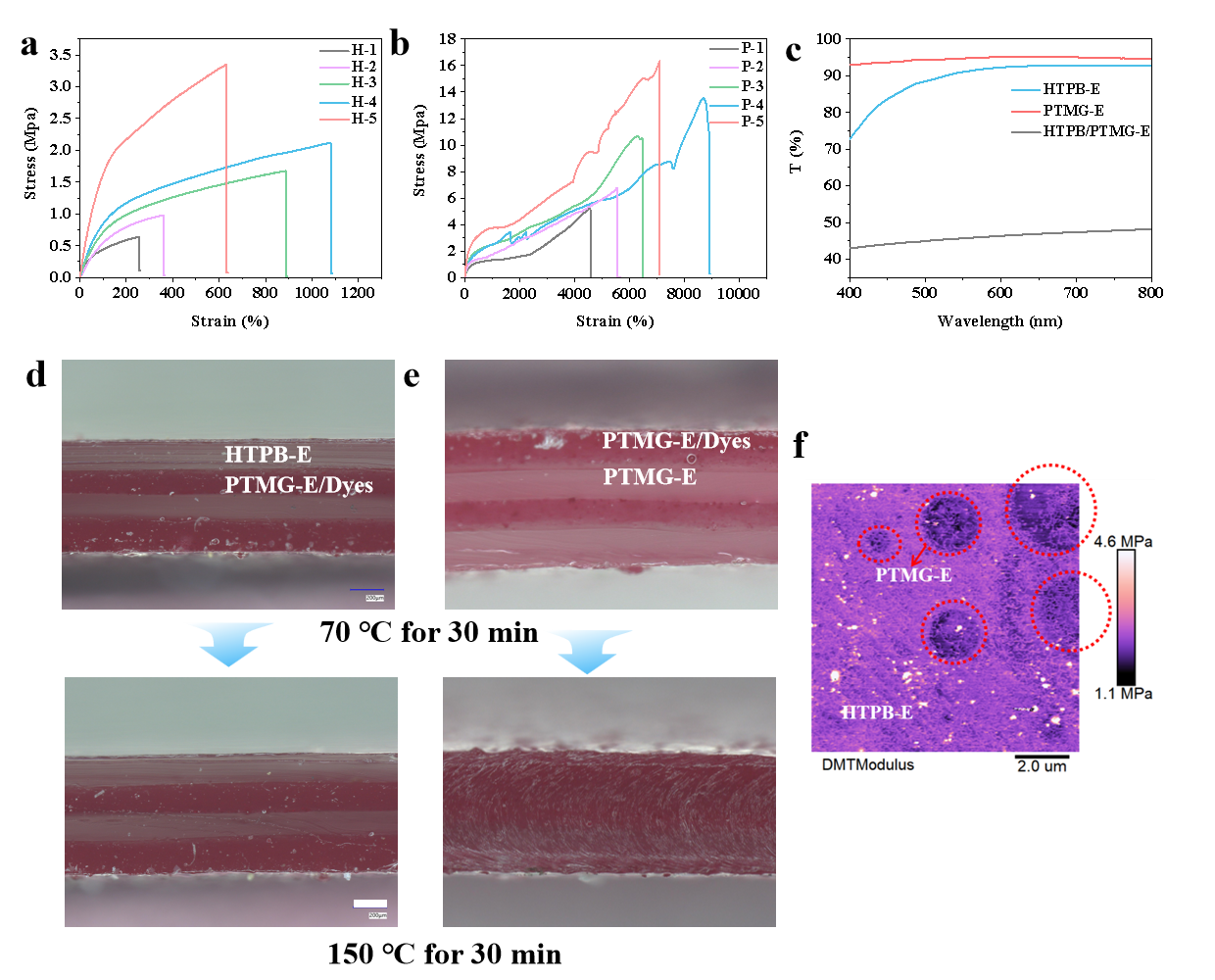


**Figure S2.** Stress-strain curves of HTPB- (a) and PTMG-based (b) elastomers with different blend ratios. (c) Transmission spectrum of PTMG-E, HTPB-E, and HTPB/PTMG-E. Photographs of heat‑treated (d) HTPB‑E/PTMG‑E/Dye and (e) PTMG‑E/PTMG‑E/Dye alternating layers. (f) AFM modulus image of HTPB/PTMG-E.

1. Optical transmittance: A blend film of PTMG-E and HTPB-E (denoted HTPB/PTMG-E) exhibits significantly reduced transmittance compared to the pure networks (Fig. S2c), indicating strong light scattering from submicron phase-separated domains typical of an immiscible blend.
2. Interfacial chain diffusion: We incorporated a trace amount of inert dye into PTMG-E (PTMG-E/Dye) and constructed two types of alternating stacks: HTPB-E/PTMG-E/Dye and PTMG-E/PTMG-E/Dye. After heating at 150°C for 30 min, the interfaces between identical PTMG-E layers became diffuse due to chain interdiffusion, whereas the HTPB-E/PTMG-E/Dye interfaces remained sharp (Fig. S2d, e). This visually proves the lack of chain mixing between the two networks.
3. AFM modulus mapping: The HTPB/PTMG-E film displays distinct high-modulus (HTPB-rich) and low-modulus (PTMG-rich) domains with clear boundaries (Fig. S2f), providing direct nanoscale evidence of phase separation. These results consistently confirm that PTMG-E and HTPB-E are immiscible networks, which provides the thermodynamic driving force for the observed alignment.

**Tables S1**. HTPB- and PTMG-based elastomers with different blend ratios.

|  | HTPB/mmol | PTMG/mmol | LDI/mmol | CBA/mmol | THDI/mmol |
| --- | --- | --- | --- | --- | --- |
| **P-1** | 0 | 1.0 | 2.0 | 1.0 | 0 |
| **P-2** | 0 | 1.0 | 2.0 | 1.2 | 0.07 |
| **P-3** | 0 | 1.0 | 2.0 | 1.4 | 0.14 |
| **P-4** | 0 | 1.0 | 2.0 | 1.6 | 0.21 |
| **P-5** | 0 | 1.0 | 2.0 | 2.0 | 0.34 |
| **H-1** | 1.0 | 0 | 2.0 | 1.0 | 0 |
| **H-2** | 1.0 | 0 | 2.0 | 1.2 | 0.07 |
| **H-3** | 1.0 | 0 | 2.0 | 1.4 | 0.14 |
| **H-4** | 1.0 | 0 | 2.0 | 1.6 | 0.21 |
| **H-5** | 1.0 | 0 | 2.0 | 2.0 | 0.34 |

**After**

**Before**

**Figure S3.** The reflection spectra of the optical devices before and after the fingers are bent.

**Figure S4.** Electrical signals from finger bending

**Figure S5.** Reflectance spectrum (a) and electrical signals (b) of the finger bending motion in the repaired device

**Figure S6.** Photographs before and after PTMG-E dissolution

**Figure S7**. Stress-Strain Curves of PTMG-E (a) and HTPB-E (b) Before and After Recycling


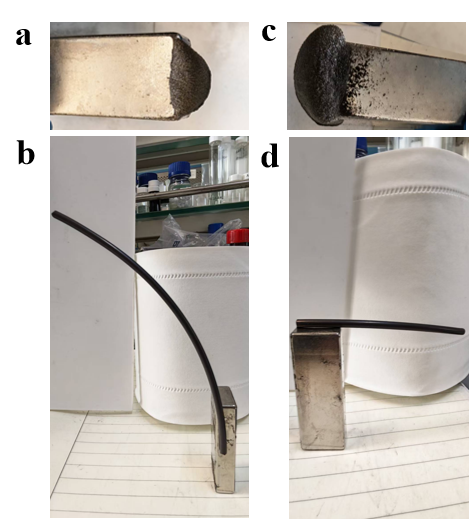


**Figure S8.** Photos of the magnetic response characteristics of NdFeB particles before (a) and after recycling (c). Photos showing the interaction between the fibrous NdFeB particles prepared before (b) and after recycling (d) and the magnet

**Figure S9.** Macroscopic electrical conductivity tests and microscopic morphology statistics (c) of carbon nanotubes before and after recycling. (a) Pristine CNTs, (b) Recycled CNTs.


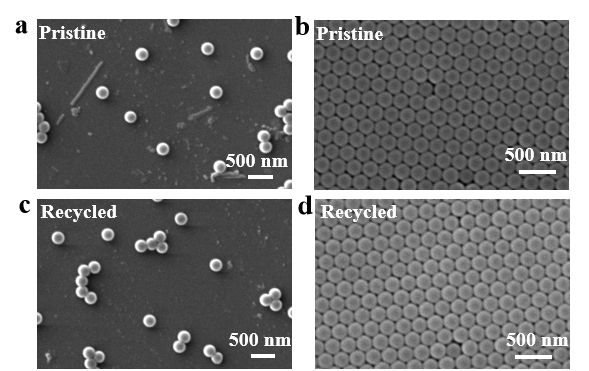


**Figure S10.** Before and after recycling, the SEM images of PS@SiO2 microspheres and the assembled products


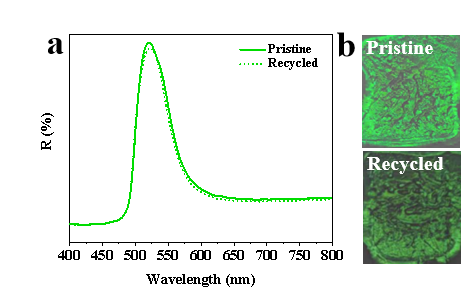


**Figure S11.** The reflectance spectrum (a) and macroscopic color (b) of the photonic crystal structure formed by the PS@SiO_2_ microspheres before and after recycling


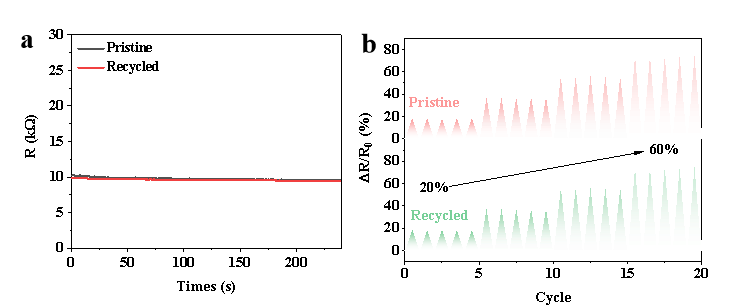


**Figure S12.** The resistance values of the CNTs-based electro-responsive sensors before and after recycling (a), as well as the response ability of the sensors based on the recycled CNTs to strain changes (b)

**Figure S13.** The stress-strain curve of recycled PTMG/HTPB-E
